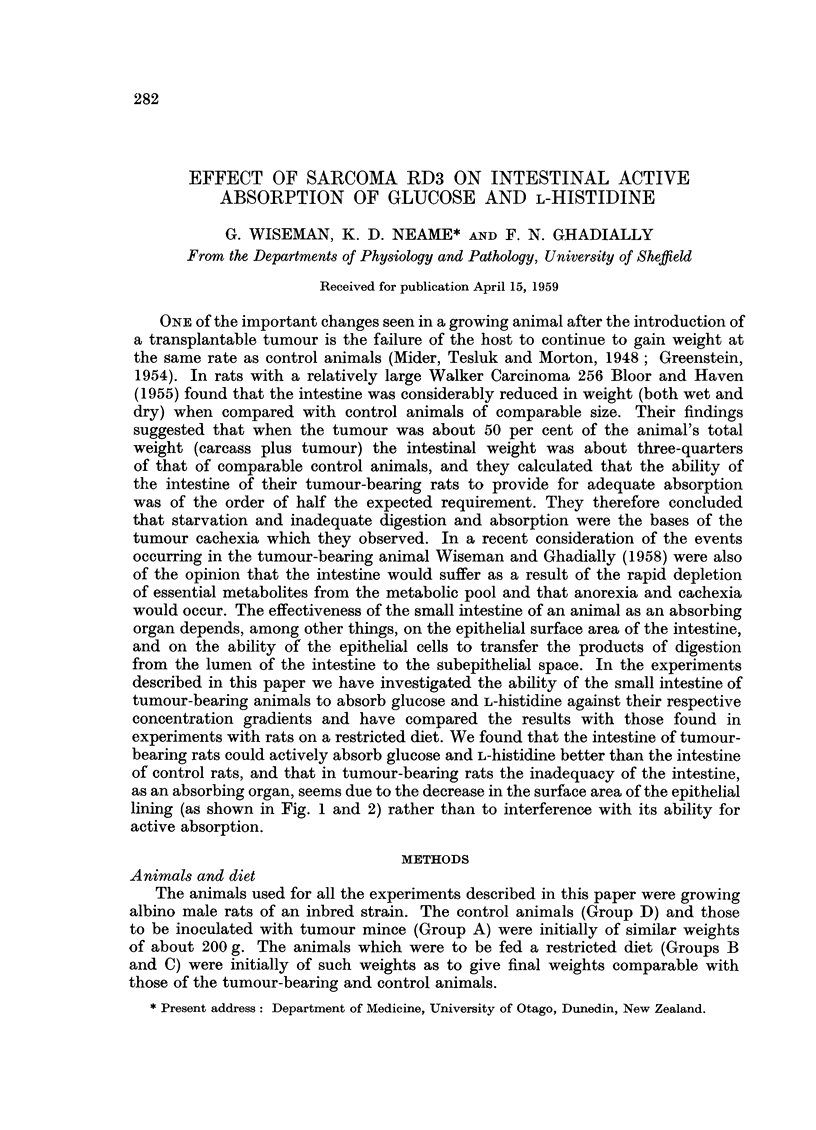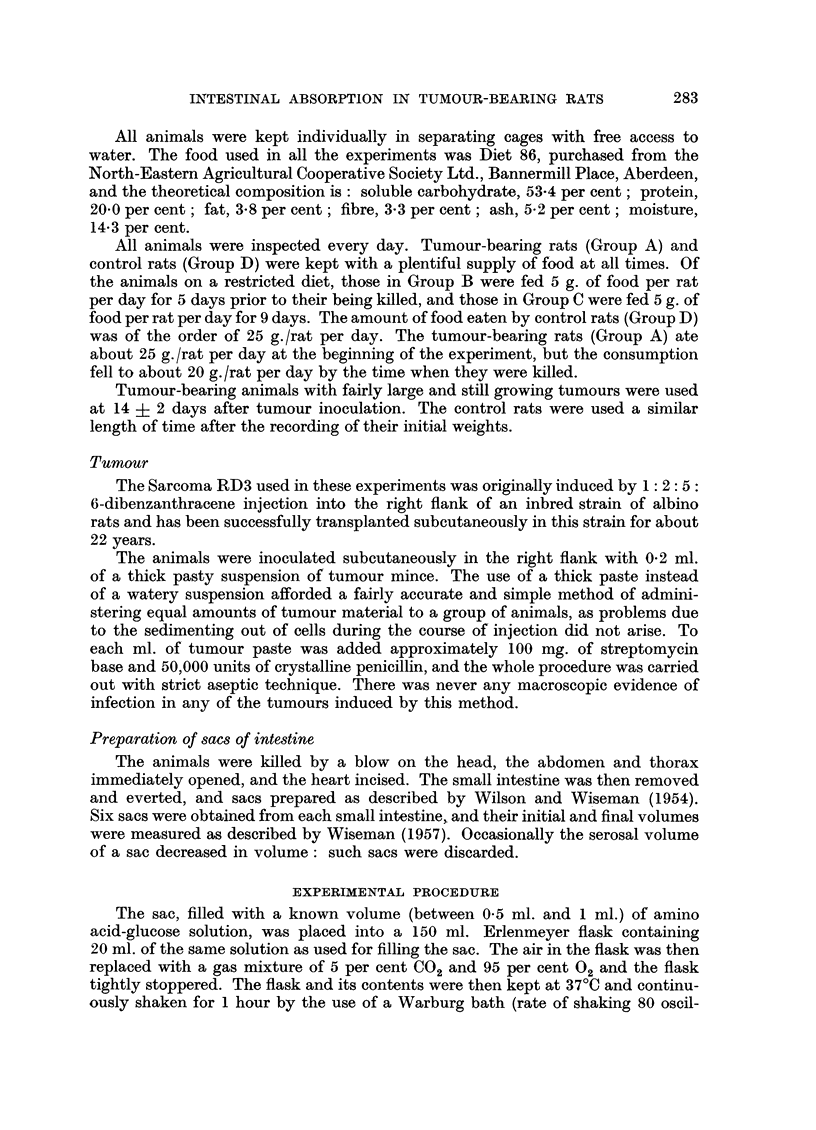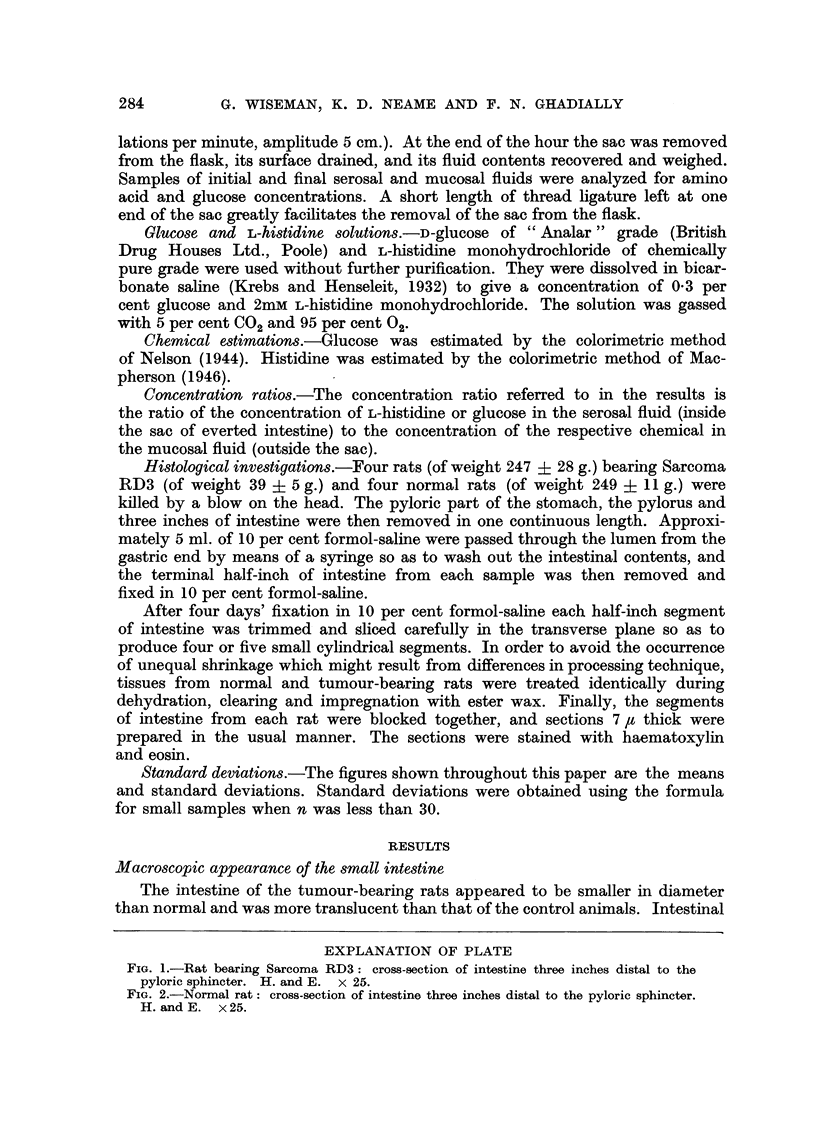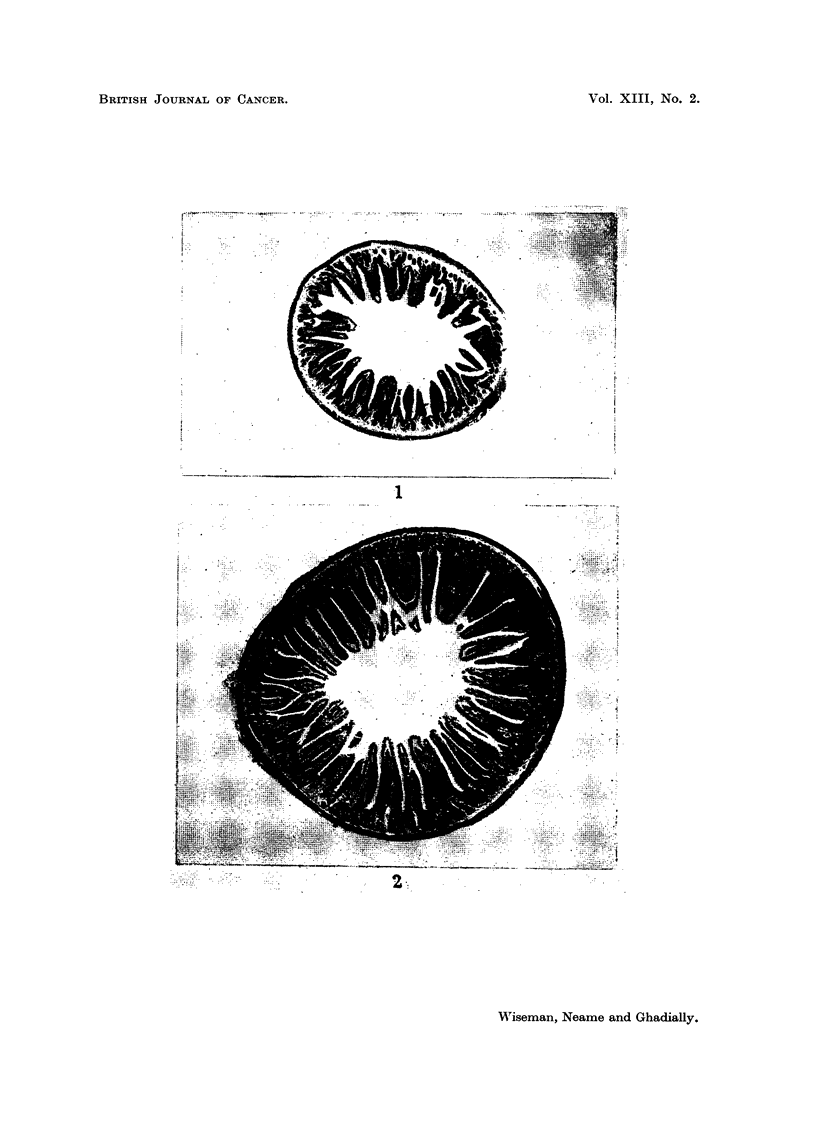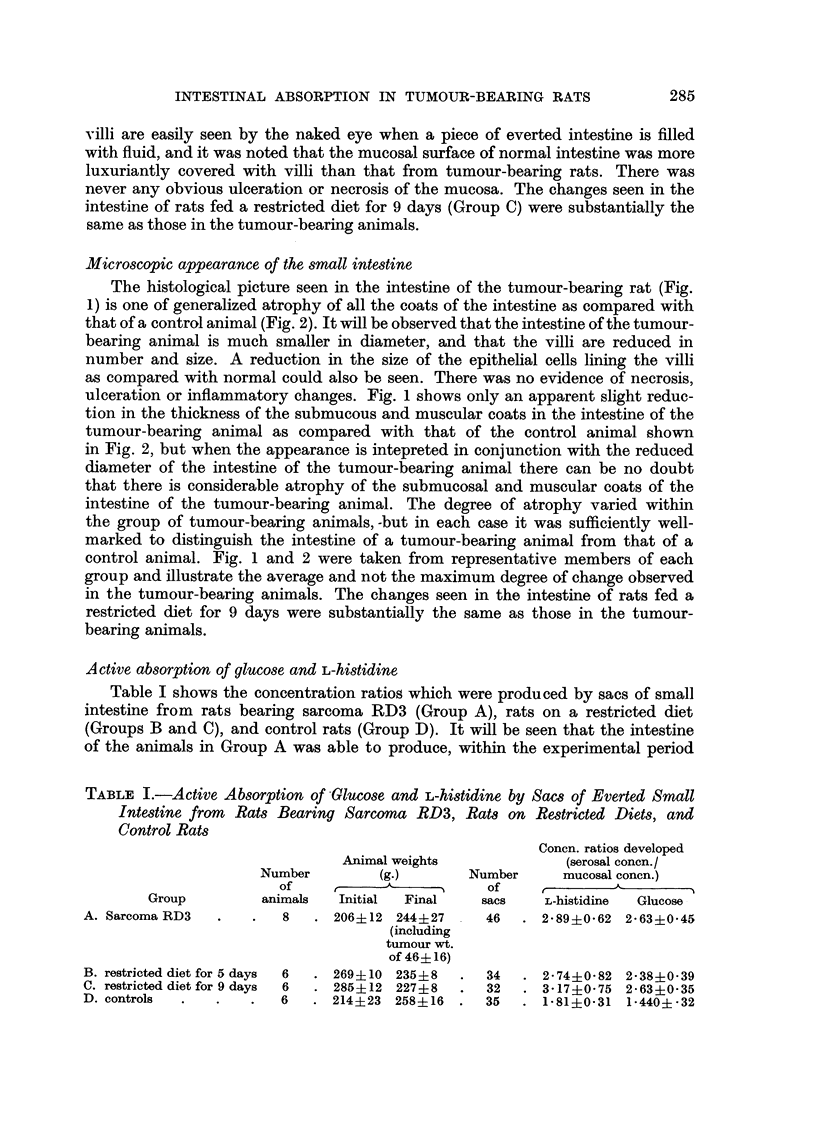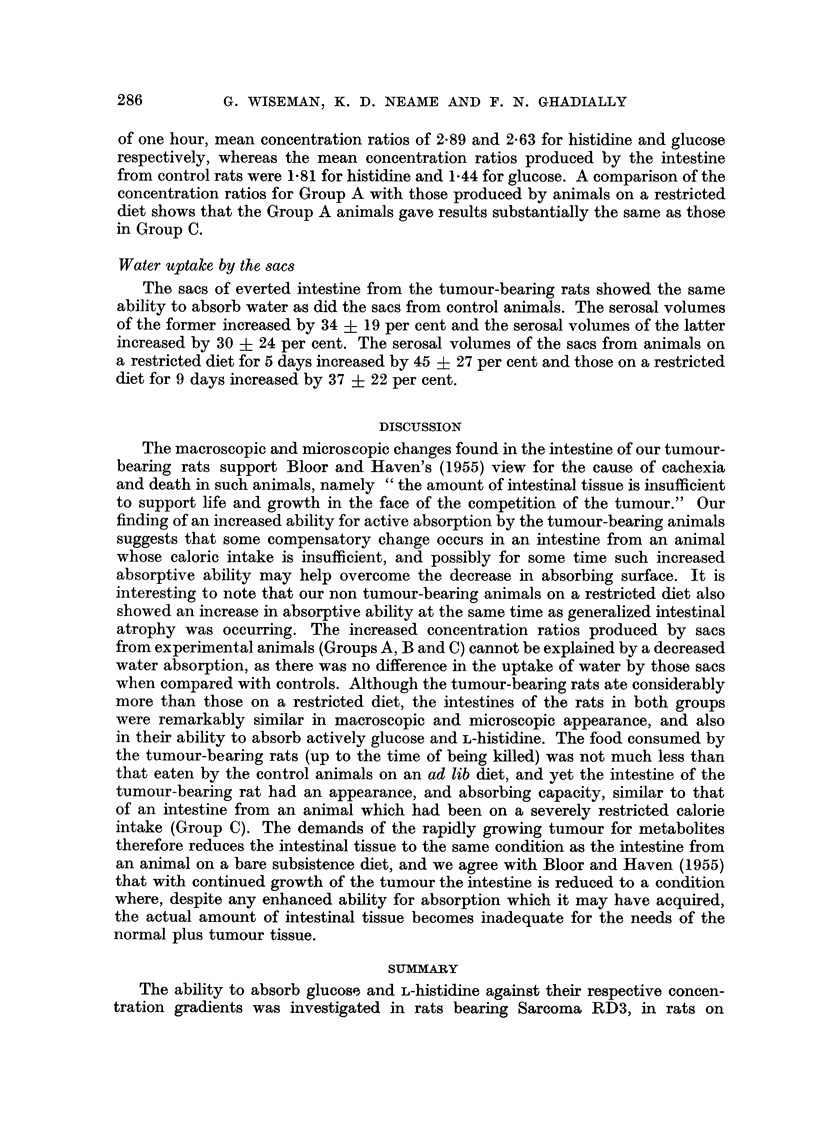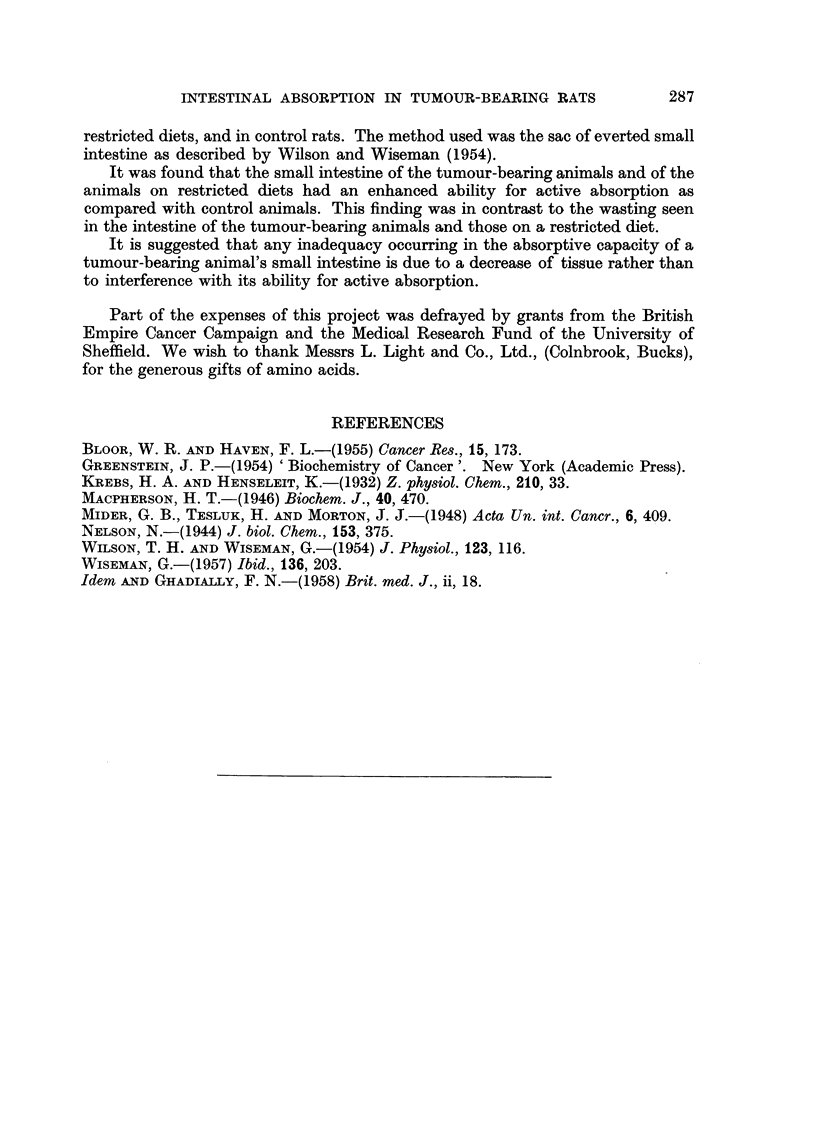# Effect of Sarcoma RD3 on Intestinal Active Absorption of Glucose and L-Histidine

**DOI:** 10.1038/bjc.1959.34

**Published:** 1959-06

**Authors:** G. Wiseman, K. D. Neame, F. N. Ghadially

## Abstract

**Images:**


### EFFECT OF SARCOMA RD3 ON INTESTINAL ACTIVE ABSORPTION OF GLUCOSE AND L-HISTIDINE

G. WISEMAN, K. D. NEAME* AND F. N. GHADIALLY

*From the Departments of Physiology and Pathology, University of Sheffield*

Received for publication April 15, 1959

ONE of the important changes seen in a growing animal after the introduction of a transplantable tumour is the failure of the host to continue to gain weight at the same rate as control animals (Mider, Tesluk and Morton, 1948; Greenstein, 1954). In rats with a relatively large Walker Carcinoma 256 Bloor and Haven (1955) found that the intestine was considerably reduced in weight (both wet and dry) when compared with control animals of comparable size. Their findings suggested that when the tumour was about 50 per cent of the animal's total weight (carcass plus tumour) the intestinal weight was about three-quarters of that of comparable control animals, and they calculated that the ability of the intestine of their tumour-bearing rats to provide for adequate absorption was of the order of half the expected requirement. They therefore concluded that starvation and inadequate digestion and absorption were the bases of the tumour cachexia which they observed. In a recent consideration of the events occurring in the tumour-bearing animal Wiseman and Ghadially (1958) were also of the opinion that the intestine would suffer as a result of the rapid depletion of essential metabolites from the metabolic pool and that anorexia and cachexia would occur. The effectiveness of the small intestine of an animal as an absorbing organ depends, among other things, on the epithelial surface area of the intestine, and on the ability of the epithelial cells to transfer the products of digestion from the lumen of the intestine to the subepithelial space. In the experiments described in this paper we have investigated the ability of the small intestine of tumour-bearing animals to absorb glucose and L-histidine against their respective concentration gradients and have compared the results with those found in experiments with rats on a restricted diet. We found that the intestine of tumour-bearing rats could actively absorb glucose and L-histidine better than the intestine of control rats, and that in tumour-bearing rats the inadequacy of the intestine, as an absorbing organ, seems due to the decrease in the surface area of the epithelial lining (as shown in Fig. 1 and 2) rather than to interference with its ability for active absorption.

### METHODS

### *Animals and diet*

The animals used for all the experiments described in this paper were growing albino male rats of an inbred strain. The control animals (Group D) and those to be inoculated with tumour mince (Group A) were initially of similar weights of about 200 g. The animals which were to be fed a restricted diet (Groups B and C) were initially of such weights as to give final weights comparable with those of the tumour-bearing and control animals.

* Present address: Department of Medicine, University of Otago, Dunedin, New Zealand.

All animals were kept individually in separating cages with free access to water. The food used in all the experiments was Diet 86, purchased from the North-Eastern Agricultural Cooperative Society Ltd., Bannermill Place, Aberdeen, and the theoretical composition is : soluble carbohydrate, 53·4 per cent ; protein, 20·0 per cent ; fat, 3·8 per cent ; fibre, 3·3 per cent ; ash, 5·2 per cent ; moisture, 14·3 per cent.

All animals were inspected every day. Tumour-bearing rats (Group A) and control rats (Group D) were kept with a plentiful supply of food at all times. Of the animals on a restricted diet, those in Group B were fed 5 g. of food per rat per day for 5 days prior to their being killed, and those in Group C were fed 5 g. of food per rat per day for 9 days. The amount of food eaten by control rats (Group D) was of the order of 25 g./rat per day. The tumour-bearing rats (Group A) ate about 25 g./rat per day at the beginning of the experiment, but the consumption fell to about 20 g./rat per day by the time when they were killed.

Tumour-bearing animals with fairly large and still growing tumours were used at 14 $\pm$ 2 days after tumour inoculation. The control rats were used a similar length of time after the recording of their initial weights.

*Tumour*

The Sarcoma RD3 used in these experiments was originally induced by 1 : 2 : 5 : 6-dibenzanthracene injection into the right flank of an inbred strain of albino rats and has been successfully transplanted subcutaneously in this strain for about 22 years.

The animals were inoculated subcutaneously in the right flank with 0·2 ml. of a thick pasty suspension of tumour mince. The use of a thick paste instead of a watery suspension afforded a fairly accurate and simple method of administering equal amounts of tumour material to a group of animals, as problems due to the sedimenting out of cells during the course of injection did not arise. To each ml. of tumour paste was added approximately 100 mg. of streptomycin base and 50,000 units of crystalline penicillin, and the whole procedure was carried out with strict aseptic technique. There was never any macroscopic evidence of infection in any of the tumours induced by this method.

*Preparation of sacs of intestine*

The animals were killed by a blow on the head, the abdomen and thorax immediately opened, and the heart incised. The small intestine was then removed and everted, and sacs prepared as described by Wilson and Wiseman (1954). Six sacs were obtained from each small intestine, and their initial and final volumes were measured as described by Wiseman (1957). Occasionally the serosal volume of a sac decreased in volume : such sacs were discarded.

### EXPERIMENTAL PROCEDURE

The sac, filled with a known volume (between 0·5 ml. and 1 ml.) of amino acid-glucose solution, was placed into a 150 ml. Erlenmeyer flask containing 20 ml. of the same solution as used for filling the sac. The air in the flask was then replaced with a gas mixture of 5 per cent $CO_2$ and 95 per cent $O_2$ and the flask tightly stoppered. The flask and its contents were then kept at 37°C and continuously shaken for 1 hour by the use of a Warburg bath (rate of shaking 80 oscillations per minute, amplitude 5 cm.). At the end of the hour the sac was removed from the flask, its surface drained, and its fluid contents recovered and weighed. Samples of initial and final serosal and mucosal fluids were analyzed for amino acid and glucose concentrations. A short length of thread ligature left at one end of the sac greatly facilitates the removal of the sac from the flask.

*Glucose and* L-*histidine solutions*.—D-glucose of "Analar" grade (British Drug Houses Ltd., Poole) and L-histidine monohydrochloride of chemically pure grade were used without further purification. They were dissolved in bicarbonate saline (Krebs and Henseleit, 1932) to give a concentration of 0·3 per cent glucose and 2mM L-histidine monohydrochloride. The solution was gassed with 5 per cent $CO_2$ and 95 per cent $O_2$.

*Chemical estimations*.—Glucose was estimated by the colorimetric method of Nelson (1944). Histidine was estimated by the colorimetric method of Macpherson (1946).

*Concentration ratios*.—The concentration ratio referred to in the results is the ratio of the concentration of L-histidine or glucose in the serosal fluid (inside the sac of everted intestine) to the concentration of the respective chemical in the mucosal fluid (outside the sac).

*Histological investigations*.—Four rats (of weight 247 $\pm$ 28 g.) bearing Sarcoma RD3 (of weight 39 $\pm$ 5 g.) and four normal rats (of weight 249 $\pm$ 11 g.) were killed by a blow on the head. The pyloric part of the stomach, the pylorus and three inches of intestine were then removed in one continuous length. Approximately 5 ml. of 10 per cent formol-saline were passed through the lumen from the gastric end by means of a syringe so as to wash out the intestinal contents, and the terminal half-inch of intestine from each sample was then removed and fixed in 10 per cent formol-saline.

After four days' fixation in 10 per cent formol-saline each half-inch segment of intestine was trimmed and sliced carefully in the transverse plane so as to produce four or five small cylindrical segments. In order to avoid the occurrence of unequal shrinkage which might result from differences in processing technique, tissues from normal and tumour-bearing rats were treated identically during dehydration, clearing and impregnation with ester wax. Finally, the segments of intestine from each rat were blocked together, and sections 7 $\mu$ thick were prepared in the usual manner. The sections were stained with haematoxylin and eosin.

*Standard deviations*.—The figures shown throughout this paper are the means and standard deviations. Standard deviations were obtained using the formula for small samples when $n$ was less than 30.

### RESULTS

### *Macroscopic appearance of the small intestine*

The intestine of the tumour-bearing rats appeared to be smaller in diameter than normal and was more translucent than that of the control animals. Intestinal

---

EXPLANATION OF PLATE

FIG. 1.—Rat bearing Sarcoma RD3: cross-section of intestine three inches distal to the pyloric sphincter. H. and E. × 25.

FIG. 2.—Normal rat: cross-section of intestine three inches distal to the pyloric sphincter. H. and E. ×25.

1

2

Wiseman, Neame and Ghadially.

villi are easily seen by the naked eye when a piece of everted intestine is filled with fluid, and it was noted that the mucosal surface of normal intestine was more luxuriantly covered with villi than that from tumour-bearing rats. There was never any obvious ulceration or necrosis of the mucosa. The changes seen in the intestine of rats fed a restricted diet for 9 days (Group C) were substantially the same as those in the tumour-bearing animals.

*Microscopic appearance of the small intestine*

The histological picture seen in the intestine of the tumour-bearing rat (Fig. 1) is one of generalized atrophy of all the coats of the intestine as compared with that of a control animal (Fig. 2). It will be observed that the intestine of the tumour-bearing animal is much smaller in diameter, and that the villi are reduced in number and size. A reduction in the size of the epithelial cells lining the villi as compared with normal could also be seen. There was no evidence of necrosis, ulceration or inflammatory changes. Fig. 1 shows only an apparent slight reduction in the thickness of the submucous and muscular coats in the intestine of the tumour-bearing animal as compared with that of the control animal shown in Fig. 2, but when the appearance is intepreted in conjunction with the reduced diameter of the intestine of the tumour-bearing animal there can be no doubt that there is considerable atrophy of the submucosal and muscular coats of the intestine of the tumour-bearing animal. The degree of atrophy varied within the group of tumour-bearing animals, -but in each case it was sufficiently well-marked to distinguish the intestine of a tumour-bearing animal from that of a control animal. Fig. 1 and 2 were taken from representative members of each group and illustrate the average and not the maximum degree of change observed in the tumour-bearing animals. The changes seen in the intestine of rats fed a restricted diet for 9 days were substantially the same as those in the tumour-bearing animals.

*Active absorption of glucose and* L-*histidine*

Table I shows the concentration ratios which were produced by sacs of small intestine from rats bearing sarcoma RD3 (Group A), rats on a restricted diet (Groups B and C), and control rats (Group D). It will be seen that the intestine of the animals in Group A was able to produce, within the experimental period

TABLE I.—*Active Absorption of Glucose and* L-*histidine by Sacs of Everted Small Intestine from Rats Bearing Sarcoma RD*3, *Rats on Restricted Diets, and Control Rats*

| Group | Number of animals | Animal weights (g.) | | Number of sacs | Concn. ratios developed (serosal concn./ mucosal concn.) | |
|---|---|---|---|---|---|---|
| | | Initial | Final | | L-histidine | Glucose |
| A. Sarcoma RD3 | 8 | 206±12 | 244±27 (including tumour wt. of 46±16) | 46 | 2·89±0·62 | 2·63±0·45 |
| B. restricted diet for 5 days | 6 | 269±10 | 235±8 | 34 | 2·74±0·82 | 2·38±0·39 |
| C. restricted diet for 9 days | 6 | 285±12 | 227±8 | 32 | 3·17±0·75 | 2·63±0·35 |
| D. controls | 6 | 214±23 | 258±16 | 35 | 1·81±0·31 | 1·440±·32 |

of one hour, mean concentration ratios of 2·89 and 2·63 for histidine and glucose respectively, whereas the mean concentration ratios produced by the intestine from control rats were 1·81 for histidine and 1·44 for glucose. A comparison of the concentration ratios for Group A with those produced by animals on a restricted diet shows that the Group A animals gave results substantially the same as those in Group C.

*Water uptake by the sacs*

The sacs of everted intestine from the tumour-bearing rats showed the same ability to absorb water as did the sacs from control animals. The serosal volumes of the former increased by 34 $\pm$ 19 per cent and the serosal volumes of the latter increased by 30 $\pm$ 24 per cent. The serosal volumes of the sacs from animals on a restricted diet for 5 days increased by 45 $\pm$ 27 per cent and those on a restricted diet for 9 days increased by 37 $\pm$ 22 per cent.

### DISCUSSION

The macroscopic and microscopic changes found in the intestine of our tumour-bearing rats support Bloor and Haven's (1955) view for the cause of cachexia and death in such animals, namely "the amount of intestinal tissue is insufficient to support life and growth in the face of the competition of the tumour." Our finding of an increased ability for active absorption by the tumour-bearing animals suggests that some compensatory change occurs in an intestine from an animal whose caloric intake is insufficient, and possibly for some time such increased absorptive ability may help overcome the decrease in absorbing surface. It is interesting to note that our non tumour-bearing animals on a restricted diet also showed an increase in absorptive ability at the same time as generalized intestinal atrophy was occurring. The increased concentration ratios produced by sacs from experimental animals (Groups A, B and C) cannot be explained by a decreased water absorption, as there was no difference in the uptake of water by those sacs when compared with controls. Although the tumour-bearing rats ate considerably more than those on a restricted diet, the intestines of the rats in both groups were remarkably similar in macroscopic and microscopic appearance, and also in their ability to absorb actively glucose and L-histidine. The food consumed by the tumour-bearing rats (up to the time of being killed) was not much less than that eaten by the control animals on an *ad lib* diet, and yet the intestine of the tumour-bearing rat had an appearance, and absorbing capacity, similar to that of an intestine from an animal which had been on a severely restricted calorie intake (Group C). The demands of the rapidly growing tumour for metabolites therefore reduces the intestinal tissue to the same condition as the intestine from an animal on a bare subsistence diet, and we agree with Bloor and Haven (1955) that with continued growth of the tumour the intestine is reduced to a condition where, despite any enhanced ability for absorption which it may have acquired, the actual amount of intestinal tissue becomes inadequate for the needs of the normal plus tumour tissue.

### SUMMARY

The ability to absorb glucose and L-histidine against their respective concentration gradients was investigated in rats bearing Sarcoma RD3, in rats on restricted diets, and in control rats. The method used was the sac of everted small intestine as described by Wilson and Wiseman (1954).

It was found that the small intestine of the tumour-bearing animals and of the animals on restricted diets had an enhanced ability for active absorption as compared with control animals. This finding was in contrast to the wasting seen in the intestine of the tumour-bearing animals and those on a restricted diet.

It is suggested that any inadequacy occurring in the absorptive capacity of a tumour-bearing animal's small intestine is due to a decrease of tissue rather than to interference with its ability for active absorption.

Part of the expenses of this project was defrayed by grants from the British Empire Cancer Campaign and the Medical Research Fund of the University of Sheffield. We wish to thank Messrs L. Light and Co., Ltd., (Colnbrook, Bucks), for the generous gifts of amino acids.

### REFERENCES

BLOOR, W. R. AND HAVEN, F. L.—(1955) *Cancer Res.*, **15**, 173.
GREENSTEIN, J. P.—(1954) ' Biochemistry of Cancer '. New York (Academic Press).
KREBS, H. A. AND HENSELEIT, K.—(1932) *Z. physiol. Chem.*, **210**, 33.
MACPHERSON, H. T.—(1946) *Biochem. J.*, **40**, 470.
MIDER, G. B., TESLUK, H. AND MORTON, J. J.—(1948) *Acta Un. int. Cancr.*, **6**, 409.
NELSON, N.—(1944) *J. biol. Chem.*, **153**, 375.
WILSON, T. H. AND WISEMAN, G.—(1954) *J. Physiol.*, **123**, 116.
WISEMAN, G.—(1957) *Ibid.*, **136**, 203.
*Idem* AND GHADIALLY, F. N.—(1958) *Brit. med. J.*, ii, 18.